# Contrast media-induced nephropathy in Tunisia: prospective case-control study with cardio-nephrological monitoring

**DOI:** 10.11604/pamj.2023.45.144.30749

**Published:** 2023-07-26

**Authors:** Meriam Hajji, Hela Jebali, Emna Chaabouni, Khadija Mzoughi, Ihssen Zairi, Sondos Kraiem, Lamia Raies, Fethi Ben Hamida, Lilia Ben Fatma, Mohammed Karim Zouaghi, Taieb Ben Abdallah

**Affiliations:** 1 Medicine A Department, Charles Nicolle Hospital, Tunis, Tunisia,; 2 Cardiology Department, Habib Thameur Hospital, Tunis, Tunisia,; 3 Faculty of Medicine of Tunis, Tunis El Manar University, Tunis, Tunisia,; 4 Laboratory of Renal Pathology LR00SP01, Charles Nicolle Hospital, Tunis, Tunisia,; 5 Nephrology Department, La Rabta Hospital, Tunis, Tunisia

**Keywords:** Acute renal failure, iodinated contrast media, risk factors, prevention

## Abstract

**Introduction:**

vascular opacification using iodinated contrast media (ICM) is often the primary diagnostic and therapeutic approach. However, the risk of post-injection nephrotoxicity of ICM is significantly higher in patients with underlying nephropathy. This study aimed to determine the incidence of Contrast Media Induced Nephropathy (CMIN) and identify predictive factors for its occurrence in patients from a cardiology department.

**Methods:**

our prospective study involved 158 patients who underwent coronary angiography or angioplasty at the cardiology department between December 2017 and May 2018. Two types of ICM were used in our study: Iopromide and Iohexol. All patients received either physiological serum (9‰) or bicarbonate serum (14‰) intravenously for hydration. We defined impaired renal function as an increase in creatinine ranging from 10 to 26 µmol/L, while CMIN was defined as an increase in serum creatinine exceeding 26.5 µmol/L. We investigated the factors associated with CMIN using logistic regression analysis.

**Results:**

the mean age of our patients was 60 ± 11 years (range: 29-82), with a predominance of men 63.9% (n=101). The most common cardiovascular risk factors were tobacco (36.1%, n = 57), diabetes (48.1%, n =76), hypertension (55%, n = 87). Pre-procedural creatinine averaged 81.1 ± 47.3 µmol / L with extremes ranging from 39 to 600 µmol / L. The median Mehran risk score was 3.2 (range: 0- 15). The interventional cardiology act consisted of coronary angiography in 86.2% (n=136) of cases, coronary angioplasty in 2.5% (n=4) of cases. We used iohexol and iopromide in 57.6% (n=91) and 42.4% (n=67) of cases, respectively. The overall incidence of CMIN was 9.5% (n=9). The multivariable regression analysis identified 4 risk factors independently linked to the occurrence of CMIN which were Pre-existing renal failure (OR: 6.05, 95%CI [1.23-29.62], p = 0.026), anemia (OR: 0.043, CI [1.03-8.96], p = 0.043), the toxic dose of PC (OR: 4.7, CI [1.28-17.7], p=0.02), and at a Mehran score = 11 (OR: 3.7, CI [0.88-15.6], p=0.036).

**Conclusion:**

the most effective approach for CMIN is prevention, which focuses on addressing modifiable risk factors to minimize the risk especially in patients with pre-existing renal failure.

## Introduction

Contrast media induced nephropathy (CMIN) is the third leading cause of acute renal failure (ARF) in hospital settings, after functional renal failure and drug-related causes [1]. It is also at the origin of the increase in length of intra-hospital stay and of a considerable additional cost. Nosologically, CMIN presents as an ARF that typically occurs between 24 and 72 hours after the ICM injection. The incidence of this complication varies depending on pre-existing risk factors and the characteristics of the ICM injection. It can reach 50% in patients with several risk factors [2]. Three international recommendations concerning the diagnosis, prevention and management of CMIN have been published: that of the European Society of Urogenital Radiology (ESUR) [3], that of the initiative Kidney Disease: improvement of Global Outcome (KDIGO) [4], And finally that of the European Renal Best Practice (ERBP) [5]. These recommendations are the result of the interpretation of studies of in various populations, most of them ambulatory. Therefore, they must be applied taking into account the particular clinical context of each patient. This study aimed to determine the incidence of CMIN, to identify predictive factors for it in a cardiology department and to assess the benefit of a protocol of hydration with bicarbonate serum compared to hydration with physiological serum, taking into account these different recommendations.

## Methods

**Study design:** this is a prospective study involving 158 patients in a cardiology department, during the period from December 2017 and May 2018 (six months). Were included patients aged over 18 years old, who underwent coronary angiography or coronary angioplasty and having medium or high risk of CMIN. Low to Medium risk was defined by an estimated glomerular filtration rate (GFR) between 45 and 60 ml/min without diabetes. High risk: GFR < 45ml/min or 45 < GFR < 60 ml / min with another risk factor. Were excluded, patients at very low risk of CMIN with GFR > 60 ml / min without other risk factors or a single risk factor without renal dysfunction and chronic dialysis patients.

**Setting:** in our study, two ICM were used, Iopromide (Ultravist 370), a low osmolarity nonionic monomer (i.e. 774 ± 8 mOsm/Kg H2O at 37°C) corresponding to 370 mg of elemental iodine per milliliter and having a viscosity of 10.6 cP at 37°C, Iohexol (Omnipaque 350), a low osmolarity nonionic monomer. (i.e. 780 mOsm / Kg H2O at 37°C) corresponding to 350 mg of elemental iodine per milliliter and having a viscosity of 9.5 cP at 37°C. We calculated two predictive ratios for the occurrence of CMIN: the volume injected / creatinine clearance ratio and the ratio of iodine dose in grams / creatinine clearance.

A toxic dose of ICM is defined by a ratio of the volume of contrast administered (ml) to the creatinine clearance in ml / min greater than two [6]. Intra-arterially, the maximum dose of iodine that can be injected is estimated by the formula: grams of iodine = Creatinine clearance in ml/min [7]. Thus, the ratio of iodine dose in grams to creatinine clearance in ml / min must be less than or equal to 1. All patients had received hydration with serum physiological at 9‰ or bicarbonate serum at 14‰ intravenously depending on the level of risk of developing nephropathy with iodinated contrast products while respecting the hemodynamic state of patients with left ventricular dysfunction in order to avoid volume overload: Low to medium risk: 45 < DFG < 60 ml / min without diabetes: 100 ml / hour six hours before the examination and 12-24 hours after. High risk: GFR < 45ml/min or 45 < GFR < 60 ml / min with other risk factor: 100 ml / hour 12 hours before the examination and 24 hours after.

**Participants:** we collected and analyzed the following data: epidemiological and clinical data: age and gender; habits: smoking; medical history: diabetes, hypertension, pre-existing nephropathy and history of ICM injection, ongoing treatments: Metformin, aminoglycosides, diuretics, blokers of renine angiotensine system..., search for abnormalities in the urine sediment by urine test with urine strips (proteinuria, hematuria…), blood pressure and hydration status, echocardiographic data: Left ventricular dysfunction is defined as a left ventricular ejection fraction < 50%. We speak of moderately reduced LVEF for a value between 40 and 49% and reduced LVEF for a value less than 40% [8].

**Laboratory analysis:** we realized for each patient, the day before the procedure: blood creatinine, urea, complete blood count. Between the 2^nd^ and 5^th^ day after the procedure: blood creatinine.

**Procedural data:** the indication of the act, the nature of the act, the state of hydration and the level of blood pressure at the time of the act.

**Variables:** chronic renal failure (CRF) has been defined as a glomerular filtration rate (GFR) less than 60ml/ min/ 1.73m^2^ of body surface area for at least 3 months. The stages of chronic kidney disease (CKD) were defined according to the KDIGO working group in 2012 [4]. The definition adopted of CMIN is that of KDIGO 2012 [4]. CMIN is defined as an increase of 25% and / or 5 mg / l (44 µmol / l) in plasma creatinemia 48 hours after injection of ICM [4]. Glomerular filtration rate (GFR): was estimated using the MDRD formula (Modification of diet in renal disease). The CMIN risk score used in our study is that validated by Mehran *et al*. [9]. The risk is considered: low if the score = 5, moderate if the score is between 6 and 10, high if the score is between 11 and 15, very high if the score ≥ 16.

**Data sources/ measurement:** the study patients were divided into 4 groups according to the filling solution and the contrast medium used: Group 1(G1): isotonic physiological serum / Omnipaque; Group 2 (G2): isotonic physiological serum / Ultravist; Group 3 (G3): bicarbonate serum 14% / Omnipaque; Group 4 (G4): bicarbonate serum 14% / Ultravist. The primary endpoint was the occurrence of CMIN defined by an increase in the absolute value of serum creatinine by 5 mg / l (44 µmol / l) or a relative increase of serum creatinine before the injection of the contrast medium by 25%, and this within 48 to 72 hours after the exam [8]. Study size: Our study population, divided into 4 groups of patients as previously defined, comprises respectively G1 (n=47), G2 (n=33), G3 (n=44) and G4 (n=34).

**Statistical methods:** data entry was performed using Microsoft Excel software, and statistical analysis was conducted using SPSS 22.0 statistical software. Qualitative variables are presented as frequencies and percentages. Quantitative variables are presented as mean, median, standard deviation (SD) with the range of values. Mean comparisons: Comparisons of two means for independent samples were conducted using the independent samples t-test. In cases of small sample sizes, the non-parametric Mann-Whitney U test was used. Percentage comparisons: Comparisons of percentages for independent samples were performed using Pearson's chi-square test. In cases where the chi-square test was not valid or for comparing two percentages, the bilateral Fisher's exact test was used. The significance threshold was set at p < 0.05. To identify risk factors directly associated with the event, a multivariate logistic regression analysis was conducted. Prior to the regression analysis, pairwise associations between the considered factors were tested. Only the factors that showed significant associations were included in the logistic regression model. Logistic regression provided adjusted odds ratios for each factor directly associated with the event, highlighting the specific role of each factor.

## Results

### General characteristics of the study population

**Figure 1:**
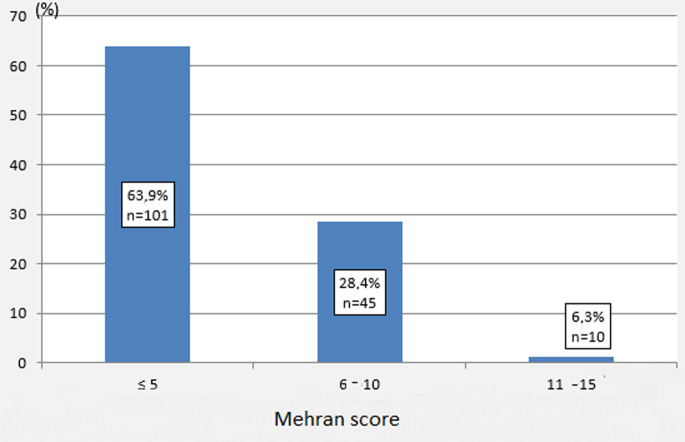
distribution of patients according to the Mehran score

The mean age of our patients was 60 ± 11 years with extremes ranging from 29 to 82 years. The age group between 50 and 69 years was the most represented with 98 patients (62%). The sex ratio M / F was 1.77. The mean pre-procedural serum creatinine was 81.5 ± 47.3 µmol / L (39 -600). The mean GFR was 91.7 ± 28.6 ml / min (8.7 - 195). The mean ejection fraction was 58.3 ± 12.6%. The rest of the clinico-biological and echocardiographic characteristics have been illustrated in Table 1. The distribution of patients according to the level of risk of CMIN according to the Mehran score is illustrated in Figure 1. The mean score in our study population was 3.2 (0 -15). No patient had a very high risk (=16). The indications of the act were distributed as follows in Table 2. The predominant cardiologic procedure including ICM injection was coronary catheterization (86.2%), followed by primary angioplasty (6.9%) then coronary angiography and ad hoc coronary angioplasty (4.4%) and last elective angioplasty (2.5%). At the time of the act the mean systolic blood pressure was 133.5 ± 19.5 mm Hg (100 -190). Fourteen patients (8.8%) presented with signs of dehydration prior to ICM injection. The mean diastolic blood pressure was 75.9 ± 12.7 mm Hg (50 -120).

**Table 1:** clinical, biological and echocardiographic characteristics of our study population

Characteristics	N (%)
Men	**101(63.9)**
Women	**57(36.1)**
History of ICM injection in less than 3 months	**10 (6.3)**
Proteinuria	**63(39.9)**
Hematuria	**38 (24)**
Chronic renal failure	**11 (7)**
Active smoking	**57(36.1)**
Diabetes	**76(48.1)**
**Hypertension**	
Anti-hypertensive treatment	**87 (55)**
BRAS	**62(39.2)**
Diuretics	**18(11.4)**
Hb<12 g/dl	**47(29.7)**
LV dysfunction	**33 (20.9)**

N: number, ICM: iodinated contrast media, BRAS: Blokers of renine angiotensine system, Hb: hemoglobine, LV: left ventricule

**Table 2:** distribution of patients according to the indications of the procedure

Indications of ICM injection	Number	(%)
ACS ST(-)	**46**	**29.1**
ACS ST(+)	**33**	**20.9**
Stable angina	**31**	**19.6**
Instable angina	**18**	**11.4**
Etiological assessment of DCM	**9**	**5.7**
Preoperative coronary angiography	**7**	**4.4**
Rhythm disturbances	**5**	**3.2**
Elective angioplasty	**4**	**2.5**
Heart failure	**3**	**1.9**
Coronary computed tomography angiography (+)	**1**	**0.6**
myocardial scintigraphy (+)	**1**	**0.6**

ACS ST(-) : Acute Coronary Syndrome without STsegment elevation, ACS ST(+) : Acute Coronary Syndrome with ST segment elevation, ICM : iodinated contrast media, DCM : dilated cardiomyopathy

**Figure 2:**
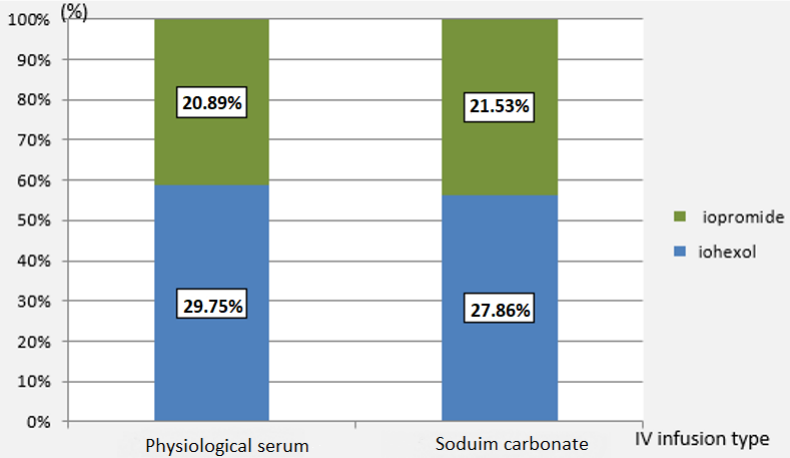
distribution of patients according to prevention measurement

We used Iohexol in 91 patients (57.6%) versus Iopromide in 67 patients (i.e. in 42.4% of cases). The mean volume of ICM administered during the angiographic procedure was 101.9 +/- 34 ml (40 -250). The volume / clearance ratio was on average 1.2 ± 0.7 with extremes ranging from 0.4 to 6.8. A toxic dose was reached in 14 patients (8.9%). The iodine dose ratio in grams / clearance averaged 0.4 ± 0.2 (0.2 to 2.4). Eighty patients (50.6%) had received physiological saline solution with an average volume of 1.7 ± 0.6 liters and 78 patients had received sodium bicarbonate (49.4%) with an average volume 1.4 ± 0.5 liter. Figure 2 shows the distribution of patients according to prevention measures.

**Figure 3:**
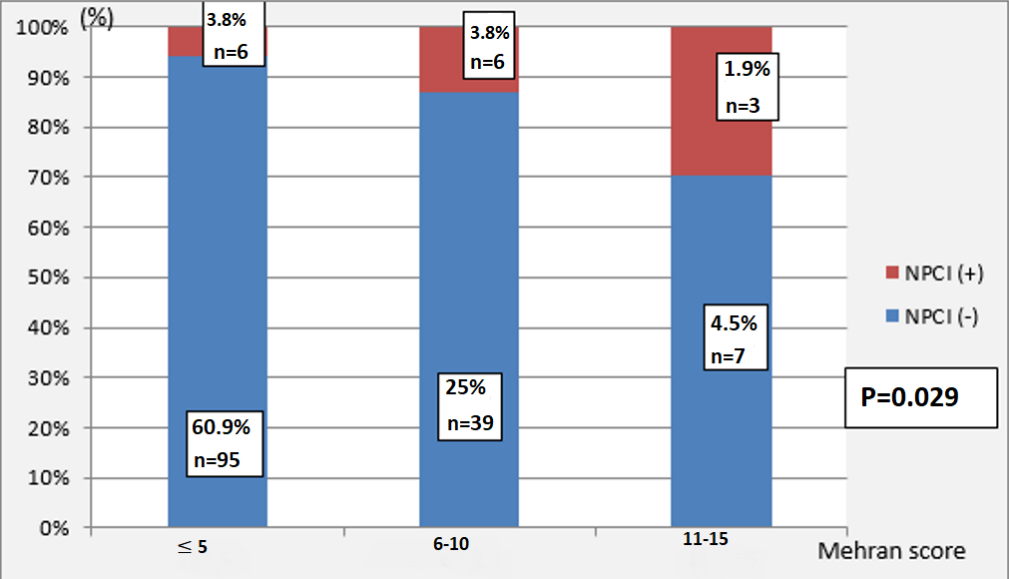
incidence of CMIN according to the Mehran score

Our study reveals that the incidence of CMIN was 9.5% (n=9). By studying the statistical correlation between the incidence of CMIN according to the medication concomitant with the injection of ICM. No medication was linked to an improvement or worsening of the CMIN risk, namely the taking of metformin, blokers of renine angiotensine system and Aminosid with respectively (p=0.35; p=0.38; p=0.74). There is also no connection between the nature of the procedure and the occurrence of CMIN. We analyzed as well in in the univariable analysis, taking into account the type of used ICM volume injected (p=0.16), volume / clearance ratio (p=0.086) and iodine dose / clearance ratio (p=0.093). Table 3 summarizes the different risk factors as we found that a significant increase in the risk of CMIN. Thus, we recorded 5.7% (n = 9) of CMIN cases for Iohexol vs 3.8% (n = 6) for Iopromide (p = 0.84). On the other hand, the incidence of CMIN in our population increased in line with increased clinical Mehran score (p=0.029) (Figure 3).

**Table 3:** incidence of CMIN and risk factors in univariate analysis in our study

Clinical characteristics	CMIN (-)	CMIN (+)	P-value
Age < 70 years old	112(70.7%)	10(6.3%)	0.17
Age ≥ 70 years old	31(19.6%)	5(3.2%)	
Male	94(59.5%)	7(4.4%)	0.14
Female	49(31%)	8(5.1%)	
Diabetes	70(44.3%)	6(3.8%)	0.5
No diabetes	73(46.2%)	9(5.7%)	
Hypertension	80(50.6%)	7(4.4)	0.49
No hypertension	63(39.9%)	8(5.1%)	
Chronic renal failure	8(5.1%)	3(1.9%)	**0.024**
No Chronic renal failure	135(85.4%)	12(7.6%)	
History of ICM injection*	0(0%)	1(0.6%)	0.23
No History of ICM injection*	143(90.5%)	14(8.8%)	
LVEF ≥ 50%	116(73.4%)	9(5.7%)	0.15
LVEF: 40-49	15(9.5%)	3(1.9%)	
LVEF < 40%	12(7.6%)	3(1.9)	
Serum creatinine level > 133 μmol/l	4(2.5%)	1(0.6%)	0.41
Serum creatinine level ≤ 133 μmol/l	139(88%)	14(8.9%)	
Anemia	39(24.7%)	8(5.1%)	**0.039**
Non anemia	104(65.8%)	7(4.4%)	
Signs of dehydration	13(8.2%)	1(0.6%)	0.75
No dehydration	130(82.8%)	14(8.9%)	
ICM Toxic Dose reached	10(6.4%)	4(2.6%)	**0.012**
ICM Toxic Dose not reached	131(84%)	11(7.1%)	

CMIN: contrast media induced nephropathy, *: less than 5 days old LVEF: Left ventricular ejection fraction, ICM: iodinated contrast media

**Figure 4:**
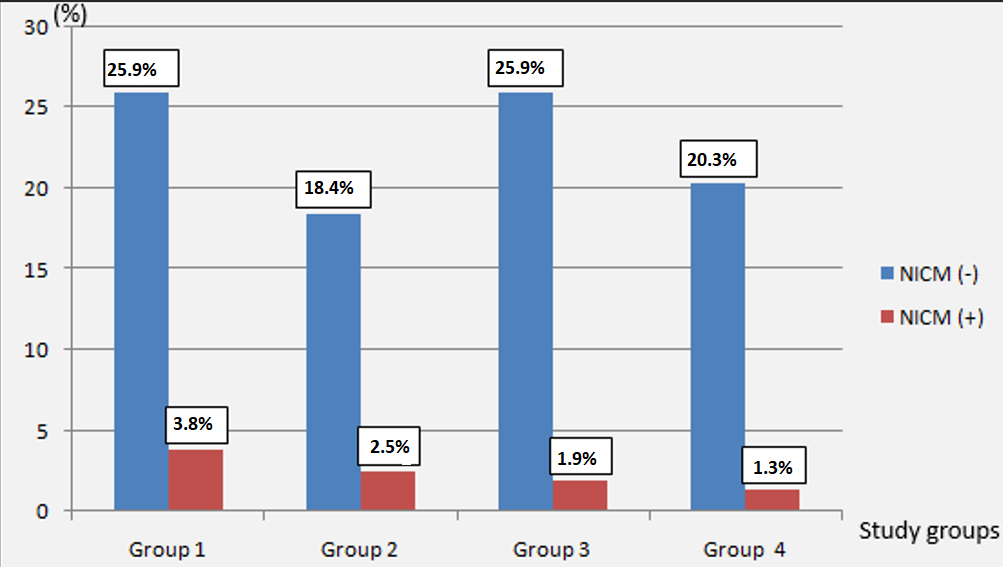
incidence of CMIN in different study groups

The multivariate analysis in logistic regression by the step-by-step top-down method identified 4 risk factors independently linked to the occurrence of CMIN which were toxic dose of ICM, pre-existing renal impairment, Toxic dose of ICM and Mehran score ≥ 11 (Table 4). The incidence of CMIN was respectively 12.7% in G1; 12.1% in G2; 6.8% in G3; 5.8% in G4. There was no significant difference in the incidence of CMIN according to the prevention protocol (p = 0.63) (Figure 4).

**Table 4:** independent factors associated with the occurrence of CMIN in our study

	Adjusted ORs (95% CI)	P-value
Toxic dose of Iodinated contrast media	4,7 [1,28-17,7]	0,02
pre-existing renal impairment	6,05 [1,23-29,62]	0,026
Mehran score ≥ 11	3,7 [0,88-15,6]	0,036

## Discussion

ICMN results mainly from medullary hypoxia induced by vasoconstriction, itself secondary to an alteration in the metabolism of vasoactive factors such as endothelin and nitric oxide. The hyperosmolality of the ICM further induces hyperviscosity, with rheological consequences resulting in superadded bone marrow hypoperfusion, aggravating the hypoxia. But above all, ICM has a direct tubular toxic effect of through the generation of free radicals. The combination of these two deleterious effects is responsible for tubular lesions which can induce necrosis of epithelial cells [10,11]. ICMN is defined as a degradation of glomerular filtration function after the intra-vascular administration of contrast media [12]. This definition is a subject of controversy and there are no universal recommendations yet [13]. However, we have retained for our study, the consensus definition of KDIGO 2012 [4].

CMIN is currently the third leading cause of hospital-acquired acute renal failure (after renal hypoperfusion and nephrotoxic drugs), accounting for 10-12% of all etiologies of ARF [14,15]. Therefore, our study aimed to determine the incidence of CMIN and identify predictive factors for its occurrence in patients from a cardiology department. Additionally, we assessed the potential benefits of a hydration protocol using bicarbonate serum compared to physiological serum. The incidence of CMIN varies between trials, ranging from 2% to 20% [16-27]. Furthermore, it is worth noting that some cases of CMIN may go unnoticed if routine serum creatinine measurements are not performed after coronary angioplasty. In our study, we observed an incidence of 9.5%, which aligns with the findings reported in the literature.

Age is known as a risk factor for CMIN [7,28] since GFR and tubular secretion decrease with age, the state of relative hypovolaemia often found in elderly patients associated with atherosclerosis of the renal arteries, as well as an underestimation of the pre-existing CRF in this group and therefore a reduced regenerative capacity of renal parenchyma after acute injury [29,30]. In our study, the mean age of the participants was 60 ± 11 years. We did not find a significant association between individuals aged over 70 years and the risk of developing CMIN (p = 0.17). Existing literature suggests that the primary risk factor for CMIN is pre-existing renal failure, with a prevalence ranging from 30% to 55% [29,30]. In our study, 6.9% of patients had pre-existing renal failure. We observed an CMIN incidence of 27.2% in this population, compared to 8.1% in patients with normal renal function (p = 0.024). Patients with diabetic nephropathy have been found to have a doubled risk of developing CMIN, with an incidence as high as 33% [31]. However, in our study, we did not observe a significant difference in the incidence of CMIN between diabetic and non-diabetic patients. This lack of difference could potentially be attributed to the beneficial effect of the hydration protocol prescribed to all of our patients. While impaired cardiac function has been associated with an increased risk of CMIN in various studies [32-34], we did not observe such a correlation in our study population. Additionally, in patients with anemia, the administration of contrast media may enhance the affinity of hemoglobin for oxygen, leading to a limited release of oxygen to peripheral tissues. As a result, local renal hypoxia could be exacerbated following exposure to the contrast agent [35,36].

Some authors have attempted to establish a risk stratification of the CMIN. Thus, different risk scores, related to patients and ICM, have been developed to predict the incidence of CMIN, the need for extra-renal purification as well as the long-term prognosis [37-41]. In our study, a Mehran score ≥ 11 was an independent factor for the occurrence of CMIN. ICMN is primarily caused by medullary hypoxia resulting from vasoconstriction, which is triggered by disruptions in the metabolism of vasoactive factors like endothelin and nitric oxide. Furthermore, ICM has a direct toxic effect on tubular cells by generating free radicals. The combined impact of these two detrimental effects leads to tubular lesions that can potentially cause necrosis of epithelial cells [42,43]. To mitigate these risks, pre-procedural hydration has been recognized as the most crucial measure [44]. By expanding the volume, hydration inhibits the renin-angiotensin-aldosterone system, reduces the release of vasoconstrictors, and diminishes the production of reactive oxygen species. Additionally, hydration helps mitigate the direct toxic effects of contrast media on the kidneys by diluting it and reducing its viscosity within the renal tubules [45]. In our study, there was no significant difference in the incidence of CMIN based on the prevention protocol (p=0.63). Five patients who received hydration with bicarbonate developed CMIN, which accounts for 3.2% of the study population. This incidence, although higher in the saline group (6.3%, n=10), did not reach the threshold of significance (p=0.19).

Strengths of our study include determining the overall incidence of CMIN in patients from the cardiology department, which was found to be 9.5%, indicating the significant association of ICM with the development of CMIN in patients undergoing coronary angiography or angioplasty. We also identified risk factors for CMIN. Our study emphasizes the importance of prevention by addressing modifiable risk factors, implementing hydration protocols for high-risk patients, and reducing the volume of contrast agents used. However, our study has limitations such as a small sample size, limited timeframe, and potential biases that may affect the generalizability of the results. Additionally, creatinine was only measured pre-procedure and on day 2-3, late disclosure of CMINs is still possible until day 10, and serum creatinine is a non-specific marker for evaluating the effectiveness of preventive measures for CMIN. Furthermore, the long-term cardiovascular and nephrological prognosis was not studied.

## Conclusion

Our study has identified a significant incidence of CMIN in patients undergoing coronary angiography or angioplasty, particularly in patients with pre-existing chronic renal failure emphasizing the importance of prevention through addressing modifiable risk factors and implementing hydration protocols. However, it warrants further research to validate our findings and assess long-term prognosis. Nonetheless, our study contributes valuable insights into CMIN and its prevention strategies based on our findings.

### What is known about this topic

- *Contrast Media Induced Nephropathy (CMIN) refers to the iatrogenic decline in renal function that occurs following the administration of intravascular iodinated contrast media (ICM);*
- *It is currently considered the third most common cause of acute renal failure acquired during a hospital stay;*
- *Established risk factors for Contrast-Induced Nephropathy (CIN) include dehydration, pre-existing renal disease, cardiac failure, and prior intravascular contrast exposure, which are associated with a high relative risk of developing CIN*.

### What this study adds

- *Our study aimed to determine the incidence of CMIN and identify predictive factors in our population;*
- *We evaluated the nephrological benefits of a hydration protocol using bicarbonate serum compared to physiological serum in patients from a cardiology department;*
- *Our study is prospective and includes comparative analysis using both univariate and multivariate methods to identify risk factors associated with CMIN*.
